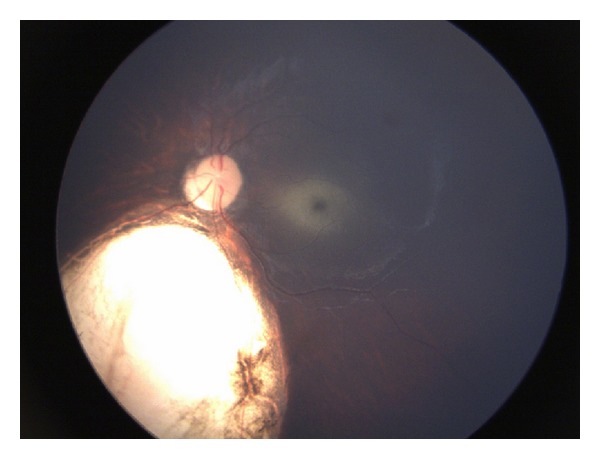# Choroidal Coloboma in a Case of Tay-Sachs Disease

**DOI:** 10.1155/2014/760746

**Published:** 2014-09-10

**Authors:** Nasreen Raees Ahmed, Koushik Tripathy, Vivek Kumar, Varun Gogia

**Affiliations:** Department of Ophthalmology, Dr. R. P. Centre, All India Institute of Medical Sciences, New Delhi 110029, India

## Abstract

Coloboma as an ocular finding has been documented in various syndromes. Here we have a case of infantile Tay-Sachs disease associated with unilateral choroidal coloboma. To the best of our knowledge, such an association has not been documented in the literature. Whether such an association is a matter of chance or signifies the involvement of ganglioside metabolism in ocular embryogenesis remains to be elucidated.

## 1. Introduction

Coloboma occurs due to failure of closure of the choroidal fissure during 5 to 7 weeks of embryonic development. It may occur as an isolated finding or have a syndromic association. Its association with various syndromes has given further insight into the pathogenesis. The association of coloboma with lysosomal storage disorders such as Tay-Sachs disease has never been documented before. Whether such an association is a matter of chance or signifies the involvement of ganglioside metabolism in ocular embryogenesis remains to be elucidated.

## 2. Case Report

A 2-year-old male child was referred from paediatrics for visual function evaluation. The child was brought by his parents with complaints that the child does not reach for objects, is unable to sit with or without support, and has generally become less playful than before.

The developmental history was suggestive of global neurological regression. On examination he was found to have generalized hypotonia with hyperreflexia. No organomegaly was present. Patient had an excessive startle response.

**Figure 1:**
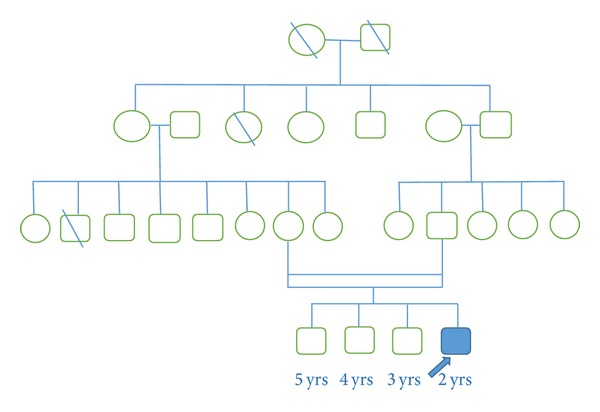


The parents gave history of second degree of consanguineous marriage. A detailed family history was taken (Figure 1). Patient had three older male siblings. The siblings were thoroughly evaluated and found to have normal neurological development as per age. There was no history of demise of any family member at a young age.

**Figure 2:**
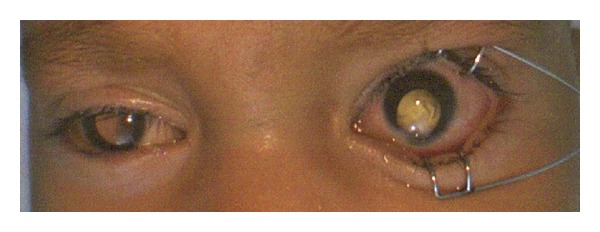


**Figure 3:**
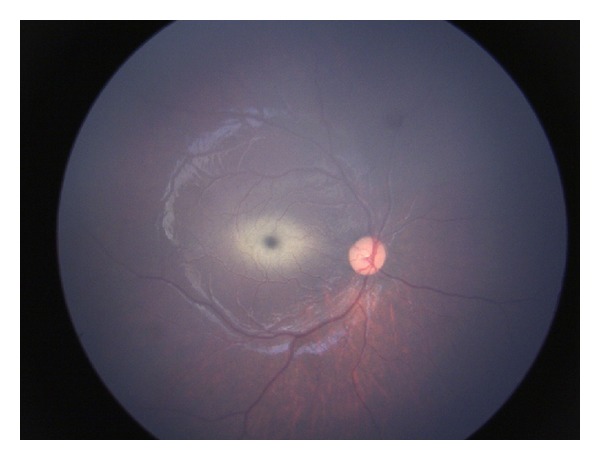


On ocular examination, pupils were reactive to light. The child did not fixate or follow light. The anterior segment in the right eye was within normal limits. In the left eye a typical iris coloboma was appreciated in the inferonasal quadrant (Figure 2). The lens in both eyes was transparent. A cherry red spot was noted in both eyes. Rest of the fundus was normal in the right eye (Figure 3). Left eye showed type III choroidal coloboma by Ida Mann's classification (Figure 4). No disc pallor was noted.

There was no evidence of nystagmus or strabismus. Refraction under cyclopentolate 0.5% was performed. Right eye had a spherical equivalent of +1.5 D and left eye had a spherical equivalent of +2.00 D. Visually evoked potential by flash stimuli showed an extinguished response in both eyes.

Blood sample sent for enzyme analysis revealed deficiency of hexosaminidase A enzyme (5.89 nmol/hr/mg). MRI scan of the brain revealed areas of hyperintensity in the supratentorial white matter, bilateral corpus striatum, and external capsule with mild cerebral atrophy. The findings were compatible with GM2 gangliosidosis—Tay-Sachs disease.

## 3. Discussion

Coloboma can occur as an isolated finding or in association with systemic features. The literature is replete with systemic associations of coloboma. While some of these associations are consistent with known genetic loci, others are of unknown etiology. Syndromes known to be autosomally dominant in inheritance include Gorlin Goltz or nevoid basal cell carcinoma syndrome [1], renal coloboma syndrome [2], Noonan's syndrome [3], Rubinstein-Taybi syndrome [4], and Crouzon syndrome [5]. Syndromes with autosomal recessive inheritance pattern include Walker-Warburg syndrome [6], Joubert syndrome [7], Bardet Biedl syndrome [8], Ellis van Creveld syndrome [9], Kartagener syndrome [10], and Meckel-Gruber syndrome [11]. X-linked recessive inheritance is seen in Lenz syndrome [12] while X-linked dominant inheritance is seen in Aicardi syndrome [13], focal dermal hypoplasia [14], and orofaciodigital syndrome [15]. Chromosomal aberrations are also associated with coloboma [16].

Ours is a case of coloboma in Tay-Sachs disease wherein there is abnormal accumulation of GM2 gangliosides in cells due to lack of a lysosomal enzyme, hexosaminidase A. This could suggest a role of gangliosides and their lysosomal metabolism in ocular morphogenesis. In a study by Blackburn et al., embryonic chick neural retina cells were incubated on surfaces adsorbed with gangliosides and a surface adsorbed with neutral glycosphingolipids, phospholipids, and sulfatide [17]. Rapid and specific cell adhesion was detected on the former. The cell adhesion varied with different purified gangliosides; GM2, GD3, and GD1 showed highest adhesion while GM1, GD1b, and GT1b showed less adhesion. This suggests a role of gangliosides in cell-cell recognition in the developing neural retinal tissue. In a study done by Dreyfus et al., there was a change in pattern of ganglioside expression with age in the retina of chick embryo [18]. Therefore any perturbation in the amount and type of ganglioside expression during embryogenesis would have profound effects on neural and retinal tissue development and differentiation. Further studies are required to identify the role played by gangliosides in ocular development.

However there is a strong possibility of occurrence of coloboma in our case to be a coincidental finding as no such association has been documented previously.

## 4. Conclusion

Research done on pathogenesis of coloboma has brought into light the genes and signalling pathways involved in ocular morphogenesis. Reporting various associations found with coloboma has not only helped to identify novel signalling pathways but also helped spearhead research into specific areas of a very complex and interconnected process of embryogenesis. The association of a lysosomal storage disorder with coloboma may be a coincidental finding in our case. Documentation of more such cases can suggest the involvement of gangliosides in ocular morphogenesis; however further research is required to expand our knowledge on the role played by gangliosides, if any, in ocular morphogenesis.

## Figures and Tables

**Figure 4:**